# Editorial: Inflammation, the link between venous and arterial thrombosis

**DOI:** 10.3389/fcvm.2024.1433858

**Published:** 2024-05-30

**Authors:** Polona Žigon, Bojing Shao

**Affiliations:** ^1^ Department of Rheumatology, University Medical Centre Ljubljana, Ljubljana, Slovenia; ^2^ FAMNIT, University of Primorska, Koper, Slovenia; ^3^ Lindsley F. Kimball Research Institute, New York Blood Center, New York, NY, United States

**Keywords:** inflammation, immunity, platelet activation, coagulation activation, thrombosis

**Editorial on the Research Topic** Inflammation, the link between venous and arterial thrombosis

## 1 Introduction

**Figure 1:**
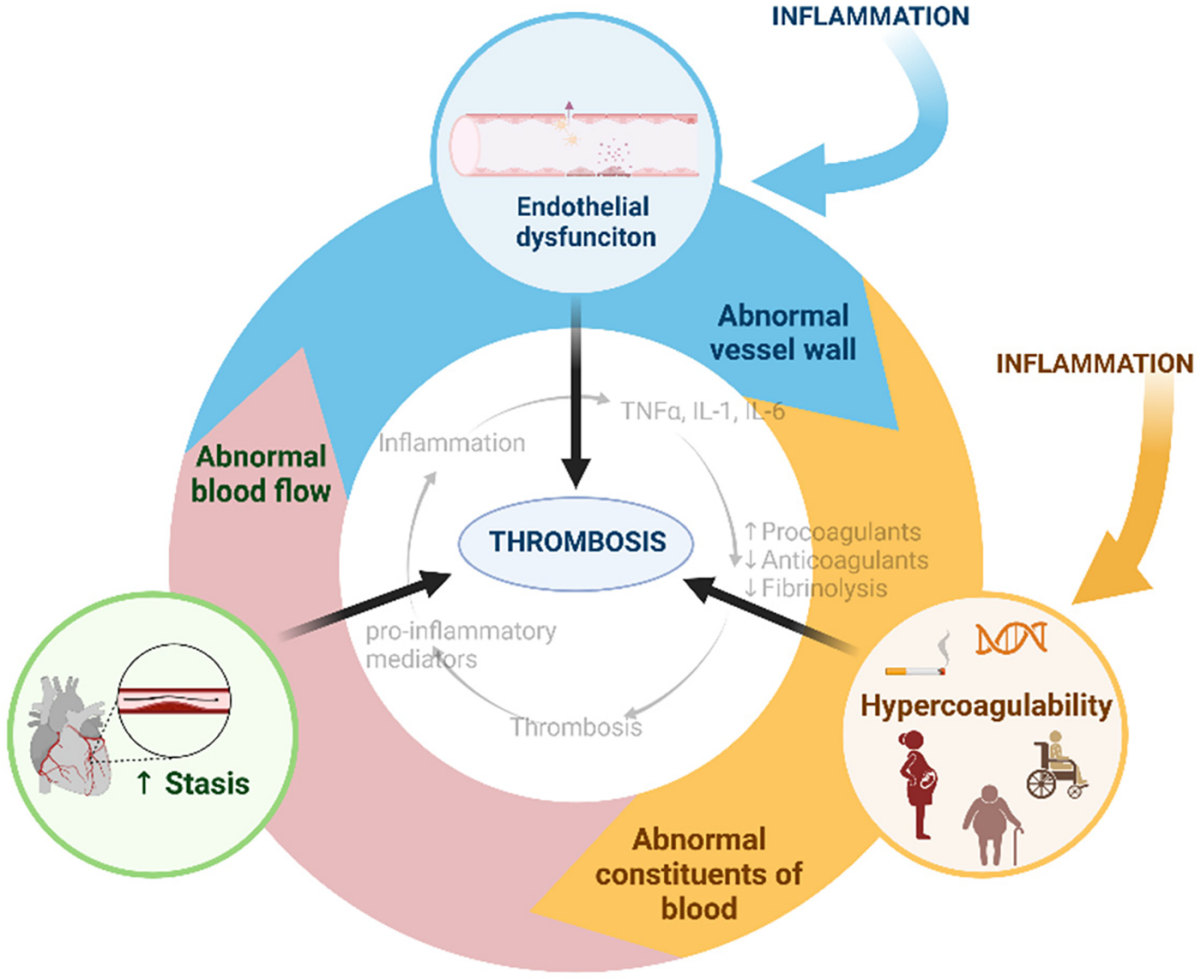
Virchow's triad for thrombosis and inflammatory associations. Created with BioRender.com.

Inflammation and thrombosis are closely linked processes that contribute to the containment of pathogen spread in an effector mechanism of host defense known as immunothrombosis. However, dysregulated or excessive immunothrombosis deteriorates tissue injury through microvascular and macrovascular thrombosis and the subsequent inflammation, termed thromboinflammation (1). The realization that inflammation stimulates thrombosis and thrombosis in turn promotes inflammation has led to an increasing recognition of the functional interdependence of these processes (Figure 1) (2). The most important factor in immunothrombosis and thromboinflammation is a vicious cycle of platelet activation and recruitment/responses of innate immune cells, which further triggers activation of the complement system and the coagulation cascade. Inflammatory conditions such as infections, chronic autoimmune diseases, and clonal hematopoiesis of undetermined potential, increase the risk of thrombotic events providing clinical evidence for the partnership of inflammation and thrombosis. Platelets and the coagulation cascade are the major players in the development of arterial and venous thrombosis, respectively. Recently, numerous studies have reported that platelets and coagulation are simultaneously involved in thrombosis during inflammation. For example, both activated platelets and increased coagulation contribute to vessel occlusion in SARS-CoV-2 infection (3–5), an acute inflammatory disease, and sickle cell disease (SCD) (6–8), a chronic inflammatory disease. Interestingly, platelets and fibrin, a marker of coagulation activation, are found in the same clot in SCD (9), suggesting that platelets and coagulation synergistically occlude the blood vessel regardless of the type of blood vessel. This possibility exists, given the complex mechanisms for thrombosis during inflammation, such as complement activation, immune response, endothelial cell injury, and stasis (1, 10, 11). Thus, inflammation can break the boundary between arterial and venous thrombosis as two distinct entities. The inflammatory components of thrombosis are a therapeutic gap and a promising target for the prevention and treatment of cardiovascular diseases such as myocardial infarction, stroke, and venous thromboembolism.

This research topic includes studies that improve the understanding of the interplay between immune cells and platelets/coagulation in inducing thrombosis during inflammation or infection, which would benefit the development of anti-thrombosis treatments under complex medical complications. Four publications focusing on the intricate relationship between inflammation and thrombosis in various clinical contexts collectively contribute to the overarching theme of our special research topic of the journal Frontiers in Cardiovascular Medicine.

## 2 Association between inflammatory conditions and deep venous thrombosis

The studies by Zhang et al., and Chen et al. provide empirical evidence and clinical insights into the association between inflammatory conditions and thrombotic events in lower extremities. Thrombus formation is a complex process, which involves the interaction of a variety of blood cells, including neutrophils, platelets and lymphocytes. Based on these blood cells, various immune-inflammation biomarkers were used as diagnostic and prognostic tools for DVT, such as neutrophil/lymphocyte ratio (NLR), and platelet/lymphocyte ratio (PLR) (12). The systemic immune-inflammation index (SII), which is a combination of neutrophils, platelets and lymphocytes, has emerged as a novel inflammation and immune marker. The SII as an integrated indicator of local immune response and systemic inflammation, has been previously reported to be significantly associated with cerebral and portal venous thrombosis, PE, and other cardiovascular diseases. Chen et al. investigated the association between SII and the risk of LEDVT in hospitalized patients. Through a comprehensive 10-year retrospective analysis, which comprised 16,725 consecutive hospitalized patients with lower extremity compression ultrasonography (CUS). They uncovered a significant link between elevated SII and an increased risk of LEDVT. Their findings reveal a non-linear relationship between SII and LEDVT risk, with elevated SII serving as a marker for heightened thrombotic risk. This study emphasizes the potential of SII as a novel inflammatory biomarker in predicting thrombotic events, shedding light on the intricate interplay between inflammation and thrombosis in clinical settings.

As is well known, thrombus originating in the lower extremity into the pulmonary arteries is considered to be the most common mechanism for pulmonary embolism (PE). Although deep venous thrombosis (DVT) and PE often differ substantially in terms of risk factors, disease presentation and clinical outcomes, DVT is believed to contribute to the occurrence of PE. Zhang et al. performed a 10-year retrospective analysis investigating whether anatomic distribution of lower extremity deep venous thrombosis (LEDVT) could be associated with an increased risk of pulmonary embolism (PE). They investigated 2,363 consecutive hospitalized patients with LEDVT of which 185 (7.83%) patients developed PE. More specifically, the proportions of PE were 5.57% for unilateral LEDVT, and 12.24% for bilateral LEDVT, the difference was statistically significant (*P* < 0.001). Regarding the thrombus location, patients with proximal LEDVT had a higher proportion of PE than those with distal LEDVT [(10.95%) vs. 6.58%, *P* < 0.001]. In conclusion they found out that patients with unilateral-proximal, bilateral-distal or bilateral-proximal are more likely to suffer from PE than those with unilateral-distal LEDVT. Despite the strengths of their study, the findings should be interpreted with some caution as this study was retrospective, single center and although the overall sample size was large, the sample size for PE were relatively small.

## 3 Occurrence of acute pulmonary embolism (PE) in adults with minimal change disease

Additionally, Rong et al. presented a case report detailing the occurrence of acute PE with arrhythmia in adults with minimal change disease (MCD). While MCD is a relatively common pathological type of nephrotic syndrome (NS) in children, its occurrence in adults is less frequent. The case underscores the potential hypercoagulable state induced by MCD, leading to thrombosis. The authors highlight the importance of considering VTE in adults with MCD, especially in the context of corticosteroid therapy, which may exacerbate the risk of blood clots. Corticosteroid is the standard of care for patients with MCD. This patient was at a significantly increased risk for VTE due to her advanced age and long-term corticosteroid therapy. This case serves as a reminder of the need for vigilance and further research into the mechanisms underlying VTE in inflammatory conditions like MCD.

## 4 Mechanistic insights into the thrombotic complications of COVID-19

In addition, the review on symmetrical peripheral gangrene (SPG) in severe COVID-19 by Wang et al. provides mechanistic insights into the thrombotic complications associated with inflammatory diseases. SPG is a commonly overlooked complication of severe COVID-19 caused by a systemic disorder rather than localized vascular disease. They have provided insights into the mechanisms and potential therapeutic approaches for SPG in severe COVID-19 cases. SPG, characterized by ischemic damage and tissue death in the extremities, is a rare but critical complication of severe COVID-19. The review explains the underlying mechanisms, including immunothrombosis, endothelial dysfunction and hypercoagulation, that contribute to the development of SPG in COVID-19 patients. COVID-19-associated SPG presented with microthrombus formation and four main features: Hypoxia, hypotension, DIC and AT depletion. Immunothrombosis, endothelial dysfunction and procoagulant platelets contribute to microvascular thrombus formation. Thrombotic microangiopathy (TMA), circulatory shock, disseminated intravascular coagulation (DIC) and anticoagulant depletion exacerbate the development of COVID-19-associated SPG. They present evidence that infection with SARS-CoV-2 causes direct damage to vascular endothelial cells, potentially inducing a pro-coagulant state. Furthermore, they report that activation of the immune system by SARS-CoV-2 infection triggers microvascular thrombosis-associated SPG and that endothelial damage and platelet activation induced by COVID-19 contributes to microvascular thrombosis-associated SPG. In addition, this comprehensive review points to the treatment strategies, which cover a broad spectrum ranging from combating the virus itself to treating the systemic effects caused by the infection. It explains the multiple treatment options, including antiviral, anticoagulant and anti-inflammatory therapies, endothelial repair and antioxidant therapies, antiplatelet and fibrinolytic therapies, plasma exchange, recovered plasma and intravenous immunoglobulins, and vasodilator therapies, and emphasizes the need for a tailored treatment regimen based on the individual patient profile and disease severity in COVID-19-associated SPG.

By examining the clinical features and potential therapeutic interventions, the review emphasizes the urgent need for further research and clinical vigilance in the management of thrombotic complications in severe COVID-19 cases.
